# Fluorescence-Based Portable Assays for Detection of Biological and Chemical Analytes

**DOI:** 10.3390/s23115053

**Published:** 2023-05-25

**Authors:** Peuli Nath, Kazi Ridita Mahtaba, Aniruddha Ray

**Affiliations:** Department of Physics and Astronomy, University of Toledo, Toledo, OH 43606, USA; peuli.nath@utoledo.edu (P.N.); kaziridita.mahtaba@rockets.utoledo.edu (K.R.M.)

**Keywords:** optical sensor, fluorescence sensor, biosensor, point-of-care, smartphone device, microfluidic

## Abstract

Fluorescence-based detection techniques are part of an ever-expanding field and are widely used in biomedical and environmental research as a biosensing tool. These techniques have high sensitivity, selectivity, and a short response time, making them a valuable tool for developing bio-chemical assays. The endpoint of these assays is defined by changes in fluorescence signal, in terms of its intensity, lifetime, and/or shift in spectrum, which is monitored using readout devices such as microscopes, fluorometers, and cytometers. However, these devices are often bulky, expensive, and require supervision to operate, which makes them inaccessible in resource-limited settings. To address these issues, significant effort has been directed towards integrating fluorescence-based assays into miniature platforms based on papers, hydrogels, and microfluidic devices, and to couple these assays with portable readout devices like smartphones and wearable optical sensors, thereby enabling point-of-care detection of bio-chemical analytes. This review highlights some of the recently developed portable fluorescence-based assays by discussing the design of fluorescent sensor molecules, their sensing strategy, and the fabrication of point-of-care devices.

## 1. Introduction

Fluorescence-based assays are among the most widely applied sensing techniques, and they have been used for a variety of applications, including biomedical research, environmental monitoring, and in the food industry, among others [1,2,3,4,5,6]. The popularity of fluorescence assays is due to their high sensitivity, specificity, fast operation, and the availability of diverse types of fluorophores that absorb and emit light covering a broad spectrum of wavelengths from ultraviolet to infrared. There are a variety of fluorescence assays—which include immunoassays, foster resonance energy transfer (FRET), fluorescence correlation spectroscopy (FCS), and polarization—that are used for sensing applications [7,8,9,10,11,12,13]. Most of these assays involve a target recognition element, commonly an antibody or a protein or chemical receptor attached to a fluorophore or fluorescence sensing agent (small molecule or nanoparticles), that bind to the specific analyte [6,14]. Upon binding to the analyte, the fluorophore often undergoes photophysical changes, resulting in variation in the fluorescence signal or the upper state lifetime, or a shift in emission wavelength, which can be detected using readout devices such as fluorometers, microscopes, or cytometers, equipped with photomultiplier tubes or CCD/sCMOS cameras [15,16,17,18,19]. These assays are highly sensitive and efficient in detecting biological and chemical analytes, but most of them are lab-based techniques that require a trained operator, dedicated workspace, involve complex steps and are time intensive. Additionally, fluorescence readout devices are bulky and expensive. Therefore, most of these assays have limited applicability in resource limited settings, as well as in the field or point-of-care (POC).

To address this issue, a significant emphasis has been placed on designing and developing low-cost, easy-to-use, portable fluorescence-based technologies, with the aim of translating measurements from the bench to onsite testing, including at the point-of-care (POC) [20,21,22,23,24,25,26]. Such onsite testing will allow for the rapid detection of analytes and biomarkers at the patient’s bedside or at home with minimal or no supervision, which will result in early diagnosis, as well as therapy monitoring, and enable physicians to build and maintain a wellness strategy remotely, without the need for patient interaction in clinical settings. Examples of some of the most widely and successfully deployed onsite biomedical testing devices include the glucometer, malaria RDT, and, most recently, the COVID test kit [27,28,29,30,31]. Similarly, environmental pollutants, such as hazardous metal ions, toxic organic compounds, and water-borne pathogens, possess human health risks, as they can be found in water, air, and food and can enter the human body through eating, breathing, and drinking [32,33,34,35,36]. Onsite monitoring and testing will facilitate the quicker elimination of these contaminants from the environment. Some examples of commercially available environment monitoring tests include fluoride, arsenic, lead test kit, air quality monitoring test etc. [37,38].

For a portable fluorescence-based assay, one of the crucial elements is the design of an assay platform that will require a small volume of a sample, for simple testing. Another important aspect is the design and development of the sensitive readout device that will detect minute changes in the fluorescence signal with high precision. Here, we focus on some of the newly synthesized fluorescent sensors; their sensing characteristics; their assay platform, along with the development of portable readout devices; and their application in healthcare, as well as environmental monitoring.

## 2. Fluorescence-Based Portable Device in Healthcare Applications

The current global healthcare crisis underpins the importance of point-of-care technologies to provide a cost-effective solution to address the unmet healthcare needs [39,40,41]. With a focus on providing rapid detection, POC devices are pivotal in containing disease, particularly those that are highly infectious. Additionally, the availability of POC devices will save time, cost, and travel for patients, particularly those who require frequent testing. This will also increase access to medical care for the underserved population [41,42]. Overall, POC devices promise to alleviate the tradeoff between high cost and poor accessibility of testing, which makes them a robust solution. Here, we explore a variety of applications related to the detection of pathogens, as well as chemical and biochemical analytes.

### 2.1. Detection of Bacterial Infection

**Figure 1:**
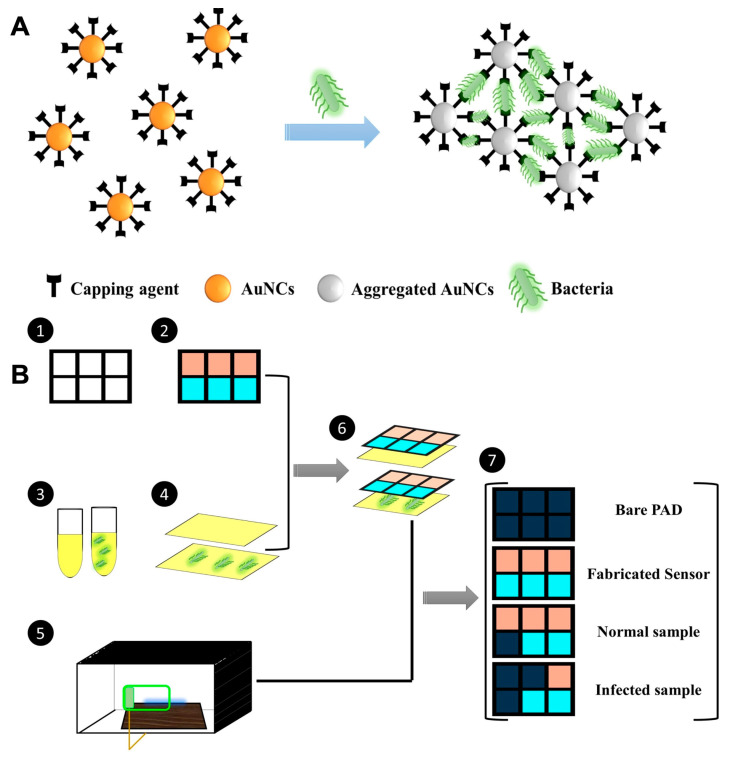
(**A**) Schematic diagram showing NCs interaction with bacteria in a sample, followed by fluorescence quenching. (**B**) Stepwise illustration of a paper-based assay system for sensing bacteria using a smartphone as a readout device for point-of-care application [54].

Bacterial infection is one of the leading causes of global mortality, owing to widespread use of antibiotics, giving rise to antimicrobial resistant strain, which has now become an emerging threat to the global healthcare system [43,44,45]. More than 2.8 million antimicrobial-resistant infections occur in US each year [45]. Some common methods of detection include bacterial culture, polymerase chain reaction, and immunoassays [46,47,48]. These methods do provide precise results but require sophisticated equipment, dedicated laboratory space, and take a long time to generate results. Many fluorescence-based portable devices have been explored in an effort to overcome the limitations of conventional lab-based techniques [49,50,51,52,53]. For example, Sheini et al. developed an ultrafast detection system for the rapid sensing of *Staphylococcus aureus*, *Streptococcus pyogenes*, *Escherichia coli,* and *Pseudomonas aeruginosa*, which are responsible for causing sepsis in children [54]. The authors developed an opto-electronic tongue (opto-E tongue) with a paper-based substrate for the effective detection of multiple bacteria species in the serum sample. The E-tongue consists of several selective fluorescent sensors, each of which has a unique response to different bacteria. The response of these sensor molecules against each bacterium species is generated in the form of a specific pattern (Figure 1), therefore making it possible to monitor and differentiate pathogenic bacterial species in a complex mixture. The sensors were based on copper (CuNCs) and gold nanoclusters (AuNCs) stabilized with proteins—pepsin, trypsin, ovalbumin, and glutathione. The detection assay, composed of six rectangular detection zones made of paper substrate (1.5 cm × 1.0 cm), was impregnated with Cu and Au NCs, which were separated by hydrophobic barrier created using an ink printing method (Figure 1). A serum sample with or without bacterial contamination was taken on a clean glass substrate and pressed on the paper sensor for interaction. The response of the sensors to each specified bacteria was monitored at an optimum pH close to serum, using a smartphone-based portable fluorometer. In the fluorometer, the paper assay device (PAD) was placed in a sample holder and irradiated with a UV light at 365 nm, as shown in Figure 1. The cabinet was equipped with a smartphone to record the fluorescence intensity change. Analyses of “before” and “after” images of each detection zone were performed using ImageJ software. The CuNCs under UV light showed a blue emission, and AuNCs showed a red fluorescence emission. After interaction with the bacterial contaminated sample, the NCs underwent fluorescence quenching. This was due to the aggregation of the NCs caused by the functional groups present on the protein shell of the NCs and the active sites of the peptidoglycan in the cell wall of the bacteria, which subsequently caused dynamic or static quenching of the NCs. Each of the CuNCs and AuNCs have a different affinity towards different functional moiety, further distinguishing between “Gram positive” (thick peptidoglycan cell wall, less lipopolysaccharide layer) and “Gram negative” bacteria (thin peptidoglycan layer, thick lipopolysaccharide layer). The limit of detection using this fluorometric PAD device was <50 Cfu/mL for *S. aureus*, *S. pyogenes*, *E. coli*, and *P. aeruginosa* (RSD < 5%). They further tested a spiked serum sample and reported a recovery percentage of 93–107%.

In another study, Xie et al., designed a point-of-care test for *Mycobacterium tuberculosis* (Mtb), using a naturally secreted enzyme by tubercle bacilli, BlaC, as a marker and BlaC-specific fluorogenic substrate, a chemically modified cephalosporin-based fluorescence molecule, as probe [55]. The detection mechanism was based on the enzymatic hydrolysis of the fluorogenic substrate by the BlaC enzyme releasing the fluorophore, thus enhancing the fluorescence signal intensity. A green-fluorescent probe—Tokyo green—was modified and coupled with cephalosporin to develop the highly stable CDG-OMe, fluorogenic substrate. In the presence of BlaC, the enzyme reacted selectively with the substrate, and there was a significant increase, more than ~200-fold, in the fluorescence intensity at 520 nm. For the practical point-of-care application, they further fabricated a simple device integrated with an LED light source, excitation and emission filters, and a smartphone. Images captured with a smartphone through the box hole made it possible to detect ~10 c.f.u live Mtb in the human sputum in less than 10 min. Therefore, this rapid TB diagnostic tool can be used for the sensitive and selective detection of low quantities of *Mycobacterium* present in the human sputum and other bodily fluids, without the need for prior sample preparation and additional complicated steps.

### 2.2. Detection of Anions and Cations

Metal ions are essential for maintaining a healthy life, as they are responsible for the regulation of cell-to-cell interaction; the proper functioning of nerve cells, the brain, and heart; muscle cells; DNA regulation; transporting oxygen; maintaining osmotic pressure; and many other biological processes [56,57]. Change in their concentrations can cause severe malfunctions in the body, e.g., growth disorders, carcinogenesis, or even death [57]. Therefore, the precise quantification of anions and cations serves as a major tool for understanding any underlying disease condition, and for maintaining a person’s overall well-being. For example, fluoride ion is an essential element that provides strength to bones and teeth, preventing dental cavities, osteofluorosis, etc. [58,59]. Common detection techniques include ion chromatography, spectroscopy, and ion-selective electrodes, all of which require complicated equipment and sample pre-treatment, making the detection process exhaustive [60,61]. Fluorescent probes, on the other hand, provide sensitive simple detection approach [62,63,64,65,66,67,68,69,70,71,72]. For “on-site” detection, Yu et al. fabricated a multicolor ratiometric fluorescent test paper for the point-of-care detection of fluoride ions (F^-^ ions) in water using filter paper [73]. The test paper (3.5 × 1 cm^2^) was prepared by an ink-printing method using “ink” made by mixing a fluoride-sensitive organic probe (C-TIPS) and F^-^ ion inert red CdTe quantum dots in optimal proportions. The fluorescent sensor showed a fluorescence “*turn-on*” effect upon binding to fluoride ions. The test paper exhibited a distinguishable fluorescence color change from red to purple to blue under a UV lamp. The probe C-TIPS was fabricated using 7-hydroxycoumarin, and the blue fluorescence of the compounds was chemically quenched by covalently conjugating it with triisopropylsilyl chloride (TIPS). Upon binding to the F^-^ ion, the recovery of the highly bright blue fluorescence suggested the removal of the TIPS moiety from the compound by cleaving the Si-O bond, thus releasing very bright blue, fluorescent coumarin in the system. The overlapping broad absorbance and excitation wavelength of the CdTe QDs, 7-hydroxycoumarin, and C-TIPS allowed the ratiometric analysis using same excitation wavelength at 365 nm. This sensor showed high sensitivity and selectivity toward F^-^ ions. In the presence of F^-^ ions, the emission peak of the sensor, at 455 nm, gradually increased, which was proportional to the concentration of F^-^ ions, while the emission peak 630 nm remained unchanged. The limit of detection was calculated to be ~0.285 μM. The practicality of the sensor was tested using lake water, tap water, well water, and human urine spiked with F^-^ ions, and showed the excellent potential of the sensor to detect F^-^ ions in a real sample, with a recovery percentage of 96.5%. The change in fluorescence color could be distinguished by the naked eye, thus allowing on-site visual assay of F^-^ ions in environmental water and biological fluids without any pre-treatment.

**Figure 2:**
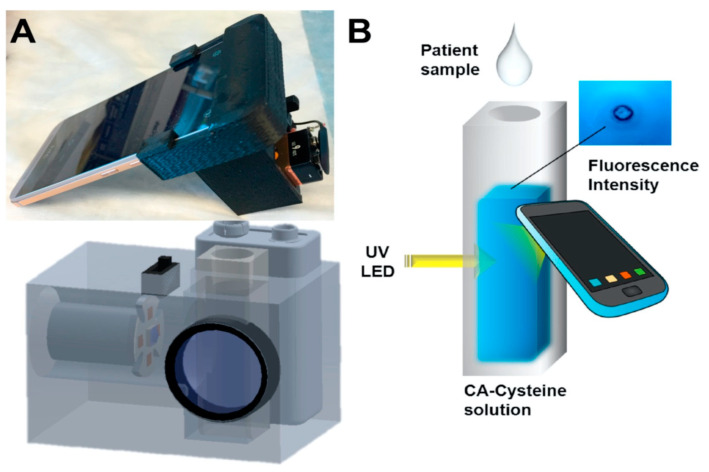
(**A**) Photo image and schematic diagram of chloridometer system. (**B**) Schematic illustration of the detection of chloride ions using a smartphone device for point-of-care application [74].

In another study, Zhang et al. established a point-of-care sweat diagnostics technique for the detection of chloride ions. They achieved this by designing a low-cost citrate-derived fluorescence sensor-based chloridometer, operated using a smartphone [74]. Citric acid (CA)-modified cysteine acted as a potential fluorescent chloride sensor to measure the chloride level present in sweat, which is an essential marker for cystic fibrosis diagnosis [74]. Apart from its cost effectiveness and high chloride selectivity, CA-cysteine was also selected as a fluorescent sensor for its exceptional photostability, high quantum yield (81%), and longer lifetime (10 ns) compared to other chloride sensors. An ultraviolet LED at 365 nm was used to excite CA-cysteine, which generated a sharp blue fluorescence signal with an emission maximum of 441 nm. The fluorescent signal was subsequently captured and measured by the smartphone camera, as shown in Figure 2. In the presence of chloride ions, the blue fluorescence signal of the CA-cysteine solution underwent fluorescence quenching, which can be attributed to the non-radiative relaxation of the excited fluorophore due to the presence of chloride ions. The detection limit was reported to be ~0.8 mM. Furthermore, successful clinical validation of the device was performed using sweat from both healthy subjects and cystic fibrosis patients.

Chromium (Cr^3+^) is another important cation in the body, and its imbalance (excess or deficiency) has been correlated to diabetes, kidney toxicity, and cardiovascular diseases [75]. Zhang et al., fabricated a hydrogel digital assay based on metal-AIEgen frameworks (MAFs) for the ultrasensitive detection of Cr^3+^ ions in water, food, and biological fluids by successfully integrating the hydrogel assay with a smartphone readout device [75]. They developed a white emissive MAFs@QDs-PVP hydrogel sensor complex by incorporating blue emissive MAFs (Ex./Em ~310 nm/465 nm) with high quantum yield ~99% and red emissive QDs (Em. 604 nm) into a polyvinylpyrrolidone (PVP) hydrogel matrix, and the combined fluorescent sensor was freeze dried to form a sponge-like hydrogel sensor. The hydrogel swells once the analyte solution comes into contact with it. The analytical performance of the sensor was tested using Cr^3+^ ions, and blue fluorescence-quenching of the MAFs was observed almost instantaneously. The limit of detection was calculated to be ~0.1 nM. In the presence of Cr^3+^ ions, the color of the white emissive sensor transitioned to red fluorescence, due to the quenching of blue emissive MAFs. Cr^3+^ ions selectively bind to the surface of the MAFs, thereby quenching the blue fluorescence of the hydrogel sensor due to the energy transfer between the Cr^3+^ ions and the MAFs, owing to their overlapping absorption and fluorescence emission wavelength. Furthermore, the sensor was tested with biological fluids such as saliva, urine, and serum, and a recovery percentage of ~103–110% was observed in spiked samples. For the point-of-care application, the images of the hydrogel sensor before and after Cr^3+^ ion interaction were captured using a smartphone and quantitatively analyzed using RGB color space. Additionally, they also demonstrated a paper-based lateral flow fluorescence immunoassay (LFIA), using MAFs for the effective detection of alpha-fetoprotein (AFP), a serum biomarker for hepatocellular carcinoma. The LFIA comprised of AFP-antibody1 (Ab1)-conjugated MAFs, a secondary Ab (Ab2) for capturing AFP, and an anti-Ab on a paper platform. Once the AFP was captured and deposited on the test line, a bright fluorescent light was observed under UV light. The estimated LOD of 0.6 pg/mL was achieved with this method. The reliability of the LFIA was evaluated using AFP-spiked human serum and with patient urine samples. The sensitivity of this MAFs LFIA was 100–1000 fold higher than traditional QDs-based LFIAs [75].

### 2.3. Detection of Biochemical Analytes

**Figure 3:**
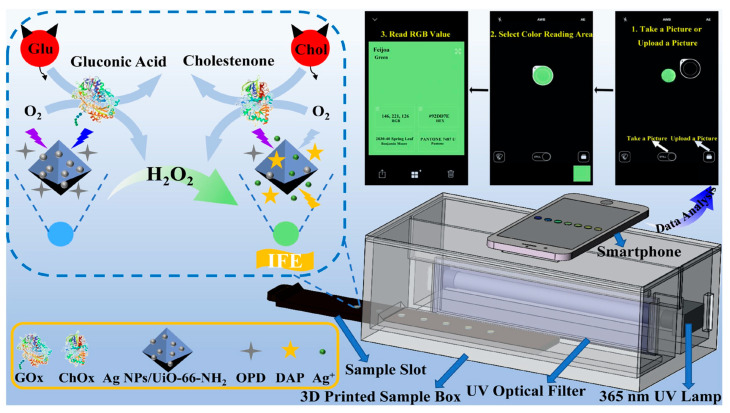
Schematic diagram of the o-Phenylenediamine (OPD) and Ag NPs/UiO-66-NH_2_ composite film with a smartphone readout device for glucose (Glu) and cholesterol (Chol) testing at the point- of-care [80].

Biochemical compounds are the building blocks of life. For example, carbohydrates, lipids, proteins, nucleic acids, and enzymes are responsible for different functions such as energy storage, acting as messengers, helping in the maintenance of cell structure, and carrying out important chemical reactions, amongst others [76,77]. An imbalance of these biomolecules can cause several life-threatening diseases, such as kidney dysfunction, jaundice, sickle cell anemia, cardiovascular diseases, hormonal imbalance, diabetes etc. [78,79]. Therefore, the precise monitoring of biochemical analytes is crucial for disease management and to maintain a healthy life. Guo et al., developed a dual emissive ratiometric fluorescent sensor molecule integrated with a smartphone for the accurate sensing of glucose (Glu) and cholesterol (Chol) in patients with metabolic syndrome [80]. The amount of Glu and Cho was determined by the quantification of H_2_O_2_, which is the principal product produced in the presence of glucose oxidase (GOx) and cholesterol oxidase (ChOx). The dual emissive sensor molecule was composed of AgNPs/UiO-66-NH_2_, where silver nanoparticles (AgNPs) acted as sensing molecules. The AgNPs were absorbed on a metal organic framework (MOF) UiO-66-NH_2_, with sodium borohydride and OPD (o-phenylenediamine) acting as a chromogenic substrate. In the presence of GOx and ChOx, the analyte glucose and cholesterol are converted to H_2_O_2_ which, in turn, etches AgNPs to release Ag^+^ ions, and further oxidizes OPD to fluorescent 2,3-diaminophenazine (DAP) in situ. Due to the overlapping emission profile of AgNPs/UiO-66-NH_2_ (Ex. 360 nm, Em. 425 nm) and the absorbance of DAP (emission 555 nm), the fluorescence intensity ratio of F_555nm_/F_425nm_ showed gradual enhancement, which was proportional to the target analyte, inducing inner-filter effect-based ratiometric sensing. The sensor showed excellent sensitivity and selectivity toward H_2_O_2_, with a detection limit of 0.2 μmol/L. As both GOx and ChOx could convert O_2_ to H_2_O_2_, the H_2_O_2_ dependent signal was translated to quantify the amount of glucose and cholesterol in the human serum samples. The quantified Glu and Cho were determined to be ~0.5 and ~0.7 μmol/L, respectively. Additionally, the fluorescence color changes from blue to yellow green are easily distinguishable by the naked eye as well. For on-site application, the sensor AgNPs/UiO-66-NH_2_, along with OPD, was immobilized on a paper substrate, which was integrated with a 3D printed smartphone attachment device, where the test strip was placed in a holder and excited by an in-built UV lamp at 365 nm, as shown in Figure 3. The captured images were processed in the RGB color space with a smartphone app. The recovery rates were found to be ~90% to 101%, using a spiked human serum sample, justifying the potential of these test strips for the quantitative analysis of glucose (Glu) and cholesterol (Chol) present in the human serum sample without any pre-treatment.

**Figure 4:**
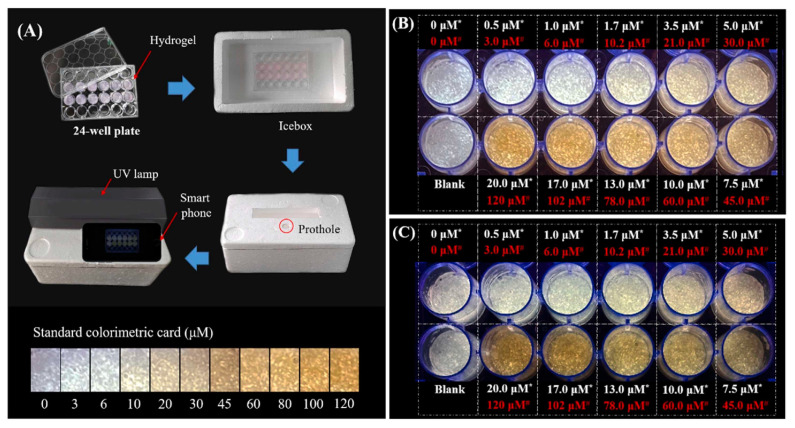
(**A**) Schematic diagram of the point-of-care testing system set-up; (**B**,**C**) the hydrogel fluorescence images after incubation with bilirubin spiked PBS buffer and urine, respectively. (Here, the hydrogel-incorporated hybrid nanosensor exhibited a white emission, while yellow represents the concentration of bilirubin in water or urine samples before mixing) [81].

In another study, Wang et al. developed a hybrid ratiometric fluorescence sensor molecule based on an aggregation induced emission (AIE) of hyperbranched polymer nanoaggregate of tetraphenylethylene (HPA-TPE) and Rhodamine B (RhB) for the selective and sensitive testing of free bilirubin in water and urine samples [81]. In patients with liver dysfunction, free bilirubin tends to get accumulated in the body, causing gallbladder disease, anemia, and neurotoxicity [82,83]. The HPA-TPE_NA_/RhB hybrid fluorescence sensor molecule was fabricated by dissolving and mixing HPA-TPE polymer nanoaggregate and Rhodamine B individually in phosphate buffer (pH = 7.4). When the system was irradiated with UV-light at 355 nm, both RhB and HPA-TPE_NA_ were excited, thereby producing dual emission peaks at 577 nm and 465 nm [81]. HPA-TPE_NA_/RhB together showed weaker emission intensity at 465 nm, compared to HPA-TPE_NA_ alone. This is due to the overlap between emissions of HPA-TPE_NA_ (465 nm) and the broad absorption peak of RhB at ~400–550 nm, causing an energy transfer between them. The addition of bilirubin exhibited a linear reduction of the fluorescence intensity ratio I_465_/I_577_ with the increment of bilirubin concentration. The blue emission, with a peak at 465 nm, was significantly suppressed due to the Förster resonance energy transfer (FRET) quenching between the bilirubin and polymer nanoaggregate, whereas the Rhodamine B emission peaked at 577 nm and did not show any sensitivity to bilirubin. This eventually shifted the fluorescence peak from blue to orange, which was observable. This mechanism resulted in a detection limit of ~25 nM in the solution phase, with recovery values in the range of ~92.5–103.3%, and relative standard deviations being less than 6.1%. They further built a portable and affordable point-of-care bilirubin detection device by incorporating xanthum gum hydrogel with this hybrid fluorescence sensor, coupled with a handheld UV light and smartphone for capturing images, as shown in Figure 4. The hydrogel showed a blue–white emission, which gradually changed to a yellow color upon increasing the concentration of bilirubin. This device was also successfully tested on human urine for quantifying bilirubin.

Enzymes are essential elements for metabolic digestion in humans. They also serve as a specific biomarker, for example, of elevated levels of trypsin or amylase, which are associated with patients suffering from pancreatitis. The normal range of trypsin in human serum is 115–350 ng/mL, an excess of which can lead to pancreatic cancer and cystic fibrosis [84,85,86]. Li et al. developed a fluorescence nano-sensing platform for the detection of trypsin (TRY) by utilizing glutathione-capped gold nanoclusters (AuNCs) [87]. Glutathione (GSH) was used as a template and stabilizer during the AuNC synthesis process. AuNCs typically have an excitation peak at 418 nm, with an emission peak observed at 625 nm. The fluorescence intensity of the AuNC was quenched by conjugating positively charged cytochrome C (Cyt C) through an electron transfer (ET) process. The catalyzing effect of TRY hydrolyzed the Cyt C, causing it to break down into negatively charged heme-peptide particles, thus weakening the ET transfer, which subsequently induced the fluorescence recovery of AuNCs. The sensor reportedly has a detection limit of ~0.08 μg mL^−1^ in a phosphate buffer solution. The practical implementation of the AuNCs-Cyt C-based platform was successfully demonstrated by analyzing TRY in spiked human serum and urine samples, with a reported recovery range between 95% and 109%, with RSD less than 4.06%. Furthermore, paper-based test strips were developed by immobilizing the AuNCs-Cyt C on absorbent papers for point-of-care detection application. The fluorescent images were captured using a smartphone and split into RGB values using ImageJ software. With the help of an image processing algorithm, a relationship between the RGB values and the logarithmic TRY concentration was established, in order to quantify the concentration.

Recently, a novel ultrasensitive fluorescence-based bioanalytical platform for the detection of multiple different biomarkers was developed [88,89,90]. In this study the authors explored nanostructures comprised of silica nanoparticles immobilized with natural NAD^+^-dependent or chimeric PQQ-dependent enzymes. The nanoconjugate was spotted on a fiberglass support. PQQ and NAD^+^-dependent enzymes are important in many cellular processes, and their activity can be used as a biomarker for various diseases. The substrate was designed to produce a fluorescent signal upon reaction with PQQ or NAD^+^-dependent enzymes via a substrate-stimulated enzymatic reduction of a fluorescence probe phenazine methosulfate or its derivatives (Ex. 365 nm, Em 465 nm). In the presence of the analyte substrate, the immobilized enzymes reacted with it, changing the color of the spot attributed to the reduced form of the dye. Images of the colored spot were captured using a smartphone camera in grayscale and processed via ImageJ software. The sensitivity was enhanced by using the multilayer formation of enzyme-functionalized on the silica nanoparticles, resulting in the amplification of the fluorescence intensity of the reduced dye in the sensor spot. The sensor platform was successfully tested on different biological fluids, such as serum, urine, saliva, and sweat, for the detection of different biomarkers, such as glucose and alcohol (LOD 10 μM), α-amylase (LOD 2 pM), and some immunosuppressive drugs (cyclosporin A and tacrolimus with LOD 2 pM).

Albumin is a protein abundantly present in the human body. Abnormal levels of albumin, especially in urine, was observed in patients with kidney disease, indicating acute kidney failure, polycystic kidney disease, vascular disease, and neoplasia, and it therefore serves as an essential biomarker [91,92]. Most of the fluorescent probes are incapable of detecting urinary albumin with precise accuracy, as urine itself displays strong fluorescence, causing significant interference with high background noise [93,94]. Zhao et al. developed a novel olefin-extended chalcone fluorescent probe DNC, with an enlarged transition dipole moment (Δμ) and an emission wavelength >600 nm, for the point-of-care detection of albuminuria in urine [95]. The red emissive fluorescent probe, DNC, far from the excitation and emission range of the urine, helped in avoiding the spectral interference from urine fluorescence. Upon excitation at 480 nm, the fluorescence from urine was negligible at 610 nm, thereby ensuring a low background signal compared to the DNC fluorescence emission in response to albumin. This eventually provided an improved and amplified signal response against albumin. The fluorescence of DNC was significantly enhanced due to binding with the specific sites (drug sites) of albumin, and it demonstrated a highly sensitive and selective determination of albumin with a detection limit of 23 nM in solution. The sensor was tested using a urine sample spiked with albumin, and it was able to detect albumin concentration of ~61 nM with RSD < 12%. An easy-to-use, smartphone-based POC system was developed for testing clinical samples collected from the patient. The mobile phone was used to capture images of the sensor, DNC, which was then analyzed using ImageJ software. The negative samples exhibited a blue color emission, while the sample spiked with albumin gradually changed from blue to a blue-violet to carmine color, which was easily distinguished by the naked eye. These results showed that the DNC probe can be successfully used to diagnose clinical A2-level and A3-level albuminuria in urine samples without pre-treatment.

## 3. Devices for Environmental Monitoring

Environmental monitoring is mainly aimed at air quality, water quality, and agriculture, with the ultimate goal of improving human health. The purpose of environmental monitoring is to identify potentially harmful microorganisms, chemicals, and other contaminants that can directly enter and pollute the ecosystem through food, air, or water [96]. For example, every year, approximately 48 million people in the US alone fall sick due to foodborne illness, among which ~3000 people die. Similarly, it has been estimated that about 35 million die due to contaminated drinking water globally, including 7.15 million from related illnesses in the US, with children being most vulnerable. Therefore, there has been a drive to developing portable testing devices for the cost-effective, real-time, on-site detection of both organic and inorganic contaminants, as well as micro-organisms, in air, water, and solid samples. This will hasten the process of detection, allowing authorities to intervene quickly in the rapid elimination of contaminants from the environment [97,98,99]. Here, we highlight some of the applications related to the detection of organic compounds, as well as parasites in drinking water.

### 3.1. Detection of Volatile Organic Compounds (VOCs)

**Figure 5:**
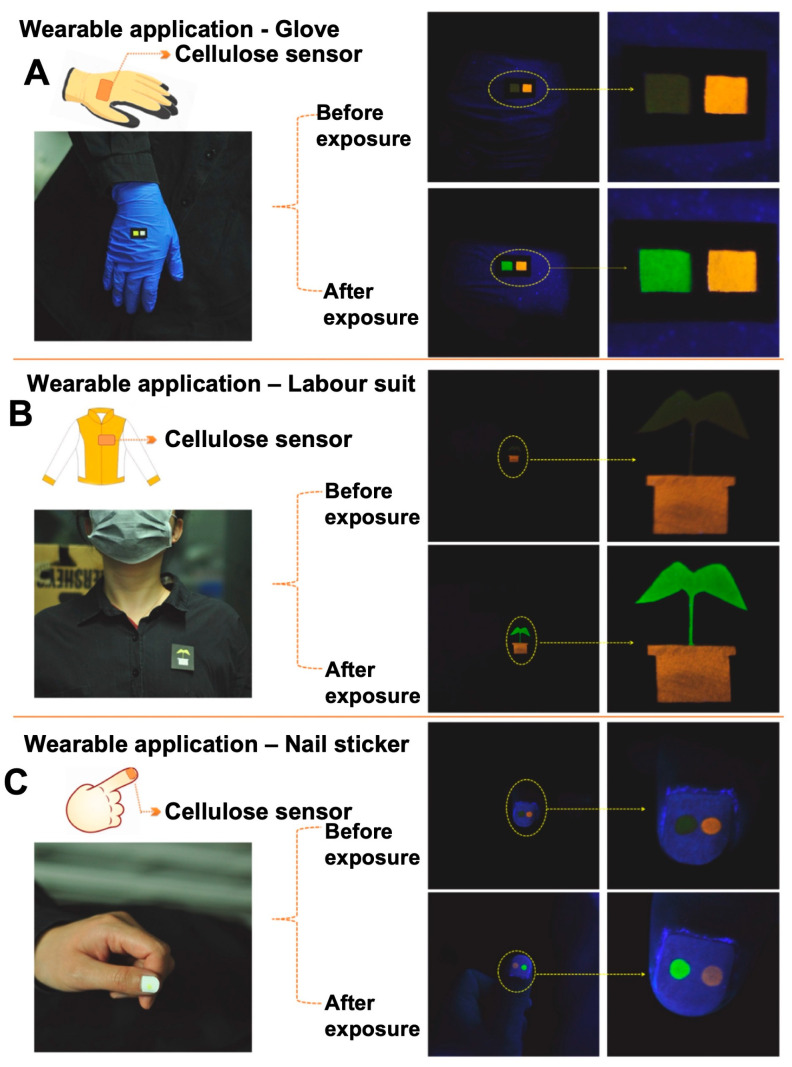
Photographs showing wearable sensor films imbedded in a (**A**) glove, (**B**) lab coat, (**C**) fingernail for the detection of VOCs in the air [101].

VOCs such as ketones, alkenes, esters, alcohols, and benzene derivatives are quite prevalent in nature, and their presence in the environment has been shown to increase health risks [34,100]. Mengyao et al. developed a donor-acceptor (D-A) luminogen HNPMO, [(E)-(3- (((2-hydroxynaphthalen-1-yl)methylene)amino) phenyl)(phenyl)methanone]-based wearable fluorescent sensor for the detection of volatile organic compounds (VOC), applying both aggregation-induced emission (AIE) and intramolecular charge transfer (ICT) strategies [101]. A film was developed by soaking the fluorescent sensor in a solid substrate such as cellulose paper, which displayed a “*turn-on*” emission upon exposure to different VOCs, such as ethyl acetate, toluene, and dichloromethane. Initially, the luminogen exhibited weak emissions due to AIE in the solid substrate, which is highly susceptible to VOCs. Upon exposure of the sensor to different VOCs, it diffuses into the molecular packing, causing intramolecular motion, exhibiting changes in their emissions via ICT. The mechanism established was the change in the disordered molecular arrangement of the sensor to a VOC-driven optimized conformation in the gas phase. The sensor exhibited either an increase or shift in emissions around ~490–523 nm at an excitation wavelength of 370 nm, depending on the polarity of the solvent. The fluorescent response to each VOC was imaged and processed using RGB and lab color space to quantify the level of VOCs. The limit of detection (LOD) of the HNPMO sensor in the presence of ethyl acetate was observed to be ~7.04 mg/m^3^ (1.8 ppm). A wearable sensor was further fabricated by drop-casting or soaking the sensor HNPMO, and an inert reference luminogen on a paper-substrate that could be easily incorporated into labor suits, gloves, and even nails, etc., as shown in Figure 5. The inert luminogen exhibited an orange emission, while the HNPMO exhibited a bright green emission upon VOC exposure for 2 min. This color emission can be easily visualized by the naked eye, and therefore it can be used as predictive tool for early warning of VOC contamination in air.

In another study, Hyungi et al. developed a multiplex analysis-based chemical sensing system, consisting of a indolizine core chemical structure called Kaleidolizine (KIz), from which a library of 75 various fluorescent KIz compounds were designed and synthesized [102]. The KIz system has been proven to possess tunable, sensitive, and predictable photophysical properties in a solid state, and it was used to build a fluorescent array on cellulose paper printed by wax. These KIz derived compounds could be excited by a single excitation wavelength of 365 nm, and lights of varying colors and intensities covering the full spectral range of visible wavelengths (416–620 nm) were emitted. Upon interaction with different chemical analytes, each Klz compound exhibited significant changes in their emission profile, influenced by the ICT process, in their molecular environment, showing intensity variations, bathochromic shifts etc. This eventually contributed to representing a unique ID for different individual analytes. A smartphone-based sensing system was developed with a phone and LED to illuminate the sensor array, which captured and processed the images using a lab-designed automated color extraction application, including various computer vision algorithms. A random forest machine-learning algorithm was trained and implemented to analyze the pattern of color changes and differentiate the detected chemicals in the sensing arrays. As a proof of concept, the system was able to detect and discriminate 35 different volatile organic compounds (VOCs) with a detection accuracy of more than 97%.

### 3.2. Water Quality Monitoring

Despite several efforts over the last few decades, more than two billion people lack access to safe drinking water. In US alone, there are 2 million people who do not have access to clean water, which makes them prone to waterborne diseases caused by bacteria, viruses, and parasites, such as giardiasis, cholera, dysentery, hepatitis etc. [103,104,105]. Koydemir et al. reported a field portable and cost-effective rapid detection platform for *Giardia lamblia* cyst, a waterborne parasite [106]. The battery-operated microscope platform consisted of a smartphone coupled with a 3D-printed opto-mechanical attachment, including a custom designed disposable cassette that holds up to ~20 mL of water. The disposable sample cassette was composed of absorbent pads, two different membrane filters (one with 8 μm pores to prevent the back flow of the particles, and one with 5 μm pores to trap the parasites). Prior to analysis, the water sample spiked with *Giardia* cyst was incubated with an anti-*Giardia* fluorescein-labelled stain and was dispensed on the capture membrane. After trapping the parasites, the cassette was then attached to the back of the fluorescent microscope, and the captured cysts in the filter membrane were then illuminated using eight blue LEDs. The images were then captured and transmitted to a server for image analysis, using a smartphone app. Machine learning was used for rapid and automatic counting, as well as differentiating of the *Giardia* cysts from other unwanted auto-fluorescence micro-objects. The limit of detection was ~12 cysts per 10 mL of water detected within 2 min after imaging with ~84% cyst-counting sensitivity. The overall turnaround time was 1 h. The group further developed a fluorescence-based portable device for the detection of microplastics [35], *E. coli* [107], live bacteria, and microorganisms in water [36,108,109,110,111].

Groundwater pollution by toxic chemicals such as antibiotic is another major problem posing a serious threat to human health [112,113]. Tetracycline is one such antibiotic overly used by humans, which is hard to degrade in living organisms and is converted into toxic compounds, causing serious groundwater pollution [114,115]. Zhang et al., constructed a ratiometric fluorescent sensor by combining molybdenum disulphide quantum dots (MoS_2_ QDs) and europium ions (Eu^3+^) to visually detect antibiotic tetracycline (TC) residues in the environment, with the help of a portable smartphone-based detection system [116]. The MoS_2_ QDs exhibited an emission peak at 470 nm, while Eu^3+^ showed a peak at 620 nm, upon excitation at 400 nm. In the presence of TC, the Eu^3+^ ion bound to TC and formed Eu-TC, with no significant shift in the emission peak, but exhibiting weak fluorescence at 620 nm through the antenna effect. However, when MoS_2_ was added, the fluorescence emission at 470 nm decreased with a significant enhancement of the fluorescence emission peak at 620 nm, due to the fluorescence resonance energy transfer between MoS_2_ and Eu^3+^ ion through the TC linear molecules, which served as a transport channel between them. Thus, MoS_2_ QDs demonstrated their potential to serve as an indicator, as well as an enhancer, in the MoS_2_-Eu^3+^ ratiometric fluorescent probe, to acquire dual signal output for TC detection. By taking the fluorescence intensity ratio at 620 nm and 470 nm (F_620_/F_470_), highly sensitive detection of TC was performed successfully in water and biological fluids with a detection limit (LOD) of 2 nM. For validation, the system was tested with a tap water sample and mouse serum spiked with TC and a recovery of 94.4–108.4% was obtained with an RSD of less than 5.36%. The transition of fluorescence color change from blue to red can be observed by the naked eye, which was translated in the development of paper-based sensing strips for “on-site” sensing. A portable readout device was developed, which was composed of a dark box, miniature UV flashlight, LED lamp, movable multifunctional filter board, and a smartphone for imaging the paper strips immobilized with a fluorescence sensor. The quantitative analysis of TC was executed by the RGB color recognition software installed in a smartphone, and a detection limit of 0.05 µM with reproducibility within a 1–40 µM TC range was exhibited.

**Figure 6:**
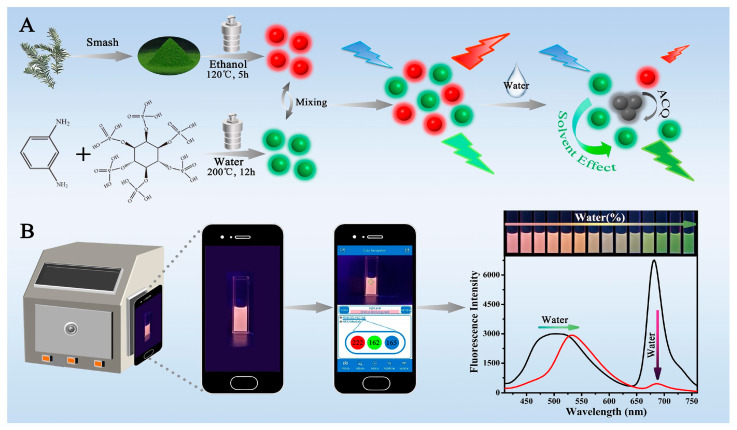
(**A**) Schematic diagram of a multicolor fluorescent probe G/R-CDs construction, (**B**) visual illustration of the portable detection of water using smartphone [121].

While water quality monitoring is important, water itself can act as an impurity in specific cases. Examples include chemical production, food processing, biomedicine, and the liquor industry, where water can lead to the hydrolysis of organic reagents, thereby degrading its quality [117,118,119,120]. Therefore, it is mandatory to monitor the water content for these applications. For this purpose, Liang et al. constructed a multicolor fluorescent sensor solution (G/R-CDs) by mixing green carbon dots (G-CDs) and red carbon dots (R-CDs) [121]. They used a solvothermal method and a hydrothermal method to synthesize R-CDs and G-CDs, respectively. Under the excitation wavelength of 390 nm, the R-CDs fluorescence spectrum exhibited an emission peak at 683 nm, with a quantum yield of 2.7%, where the fluorescence intensity gradually decreased with the increment of water content via an aggregation-caused quenching (ACQ) effect. However, the emission peak of the G-CDs experienced a red shift from 500 nm to 535 nm with a 7.9% quantum yield due to the solvent effect, while the overall fluorescence intensity remained unaffected with the addition of water content. As a result, G-CDs were used as the reference signal and R-CDs as the response. A linear relationship was established between the fluorescence intensity ratio of F_535_/F_683_ and the water content in two different ranges of 3–30% and 40–60%, and a detection limit of 1.2% was obtained. In this system, G-CDs served as toners to enhance the visualization effect of G/R-CDs by complementing their fluorescence color with an exhibition of different colors, such as orange, pink, green, and grey, with individual water contents. The color variation was easily distinguishable with the naked eye; however, to eliminate human error, a smartphone was used to capture images and split the images into RGB values of the fluorescence color [121], as shown in Figure 6. This method was successful in detecting water in commercial liquor, medical alcohol etc., with an RSD value of less than 5%, and it thus manifested its potency to serve as a visual portable detector in the field of analytical chemistry.

## 4. Typical POC Fluorescence Readout Devices

Fluorescence readout devices are a crucial aspect of POC assays, as they enable quantitative analysis of the biochemical analyte of interest. Particular emphasis needs to be placed on device design, in order to make them lightweight, compact, and low-cost. The readout devices generally consist of light sources to excite the sample; optical bandpass filters to spectrally separate the excitation and emission wavelengths; lenses for focusing the exciting light and for collecting the fluorescence photons; and, finally, a detector to record the fluorescence signal [22]. Of the many types of light sources available, diode lasers are more commonly used in POC devices. Other types of light sources that have been explored include flashlights and high-power LED lights [122]. Depending on the applications, it may be necessary to include multiple light sources for multi-spectral imaging. Incorporating multiple light sources can provide flexibility and versatility for measuring different types of fluorescent molecules, which is needed for multiplexed or ratiometric assays [123]. Sometimes heat sinks are also used to control the temperature of the light sources, in order to maintain stable performance and minimize fluctuations in output power. For detecting fluorescent photons, most of the POC devices use complementary metal oxide semiconductors (CMOS) cameras, such as smartphones, photodiodes etc. Other detectors, such as low-cost charge-coupled devices (CCDs), photodiodes (PDs), and avalanche photodiodes (APDs), have also been explored [22,124,125,126]. Advanced detectors, such as superconducting nanowire single-photon detectors, which have extremely high sensitivity and low noise levels, can be explored for even more precise and sensitive fluorescence detection [127]. Additionally, modified CMOS sensors for imaging at near infra-red wavelengths can also be beneficial, as they can avoid issues related to background fluorescence, including autofluorescence [128]. All these components need to be housed together in a compact lightproof box, which can be easily constructed using 3D printing. With the use of computer-aided design (CAD) software, the 3D model of the device can be created and then printed cheaply. Furthermore, 3D printing can also be used to create a variety of components, such as the sample holder, microfluidic channels, and the holder of optical components, as the 3D printer can be customized to specific requirements [122,129]. The design of the sample holder is important in order to facilitate easy integration of the assay platform, e.g., the microfluidic channels, to enable precise control of sample delivery and mixing.

While many portable fluorescence sensing systems still rely on external instruments to aid in their operation, such as a laptops or computers connected to controlling and processing the data, single-board computers like Raspberry Pi or Arduino offer a solution to this problem. Raspberry Pi computers are compact, low-cost, and require low power, making them ideal for portable applications [130,131]. They can be used to handle complicated tasks such as image processing, data analysis, and controlling peripheral devices, simultaneously. Alternatively, the Raspberry Pi can be integrated with a smartphone, controlled, and accessed with a smartphone app. The Raspberry Pi can even control the smartphone itself, sending signals to its built-in sensors, or triggering actions based on data from the smartphone’s camera or GPS. Another way to connect Raspberry Pi and smartphones is through wireless communication protocols such as Bluetooth or Wi-Fi. For instance, the Raspberry Pi can be configured as a Wi-Fi hotspot, allowing smartphones to connect to it and access its resources or services, thereby facilitating data transfer and processing in real-time.

## 5. Conclusions

The present review paper highlights some of the recently developed fluorescence-based portable assay techniques for the sensitive and effective detection of biochemical analyte, pathogens, anions, and cations, as summarized in Table 1. These fluorescence assays were integrated into paper-based platforms or hydrogels and coupled with smartphone-readout devices or even wearable optical sensors. Although fluorescence-based assays are becoming widely popular for the point-of-care detection of important biological and environmental analytes, some challenges lie ahead, which include improving the signal-to-noise ratio, multiplexing, and expanding its utilization for a wide variety of analytes. One specific way of increasing the fluorescence intensity is by utilizing surface plasmon resonance. This can be achieved by integrating plasmonic (typically gold or silver) nanoparticles or substrates with the assay platform [126,132]. This approach has been shown to enhance the fluorescence signal manyfold, and can also be easily integrated with POC devices [133,134,135,136,137]. Additionally, the advantage of using plasmonic nanoparticles is that they enable the colorimetric sensing of biomarkers. A few of the plasmonic NP-based tests are already available commercially, e.g., pregnancy tests and COVID test kits. Various strategies have been employed for signal amplification in colorimetric assays to achieve nanomolar to picomolar level detection, such as the detection of bilirubin using gold nanocages (LOD ~39.35 nM), or trypsin using peptide-immobilized AuNPs (LOD 0.5 nM) [138,139,140]. For further improvement of colorimetric assays to the sub-picomolar detection level, different strategies have been applied [141,142]. For example, it has been shown that the sensitivity of plasmonic NPs can be enhanced by modulating their signal intensity. This method includes the acquisition of multiple images of the test and control lines using two distinct wavelengths, ~525 nm (which corresponds to the peak absorption of nanoparticles) and 660 nm (where there is no nanoparticle absorption)—at a frequency of 5 Hz, which resulted in the periodic flashing of the test line. This approach led to ~58-fold improvement on conventional LIFA readout devices, enabling the detection of sub-200 fM concentrations [142]. Modulation of signal intensity has been used to enhance the signal of multiple modalities, including fluorescence and holography [143,144]. Similar modulation-based strategies can be employed in the POC fluorescence readout, as well as to improve the limit of detection by increasing the signal-to-noise ratio. Another way to improve sensitivity, is with the use of novel fluorescent nanomaterials, which has excellent photostability and a high fluorescence quantum yield (>90%), e.g., quantum dots, polymer dots, carbon dots etc. [145,146,147,148,149,150,151,152,153,154,155]. Another area of improvement is the assay platform, for example by utilizing novel microfluidic chips that enable testing with ultrasmall sample volumes [129,156,157,158,159,160]. The microfluidic chips can also be used for filtration and sample purification, which can result in higher accuracy [129,161,162]. Another crucial challenge at present is the miniaturization of these assay platforms and detection systems in order to expand their utility, particularly with regard to wearable devices (band, rings, patches, watches, glasses, shoes) [163,164,165,166,167]. Presently, wearable devices are very popular, as they make it possible to monitor a person’s vitals in real time [168,169]. These devices are often embedded with the Internet of Things (IoT) for the easy transfer of real-time data to physicians, thereby helping them with diagnostics and the decision-making process [170,171,172,173]. An IoT-enabled time-resolved fluorescence reader for the POC detection of *Plasmodium* antigen was recently reported [174]. However, the application of IoT in fluorescence-based POC devices is yet to be properly realized. We believe that IoT-enabled sensitive fluorescence-based POC devices will have a great impact in aiding personalized medicine by enabling testing at home or at the point-of-care. These low-cost devices can be used globally and have the potential to alleviate the huge healthcare cost in both developed and under-developed countries.

## Figures and Tables

**Table 1:** A summarized list of fluorescence-based portable sensors for the detection of some infectious pathogens and chemical analytes.

Analyte	Detection Platform	Detection Device	LOD	References
Bacteria *S. aureus*, *S. pyogenes*, *E. coli*, *P. aeruginosa*	Paper-based	Smartphone	<50 Cfu/mL	Sheini et al. [54]
*Mycobacterium tuberculosis* (Mtb)	Solution	Smartphone	10 c.f.u	Xie et al. [55]
Fluoride ions	Paper-based	Naked eye	0.285 μM	Yu et al. [73]
Chloride ions	Solution	Smartphone	0.8 mM	Zhang et al. [74]
Chromium ion	Hydrogel	Smartphone	0.6 pg/mL.	Zhang et al. [75]
Glucose and Cholesterol	Paper-based	Smartphone	0.2 mmol/L (H_2_O_2_) 0.5 mmol/L (Glu) 0.7 mmol/L (Cho)	Guo et al. [80]
Bilirubin	Hydrogel	Smartphone	25 nM	Wang et al. [81]
Trypsin	Paper-based	Smartphone	0.08 μg/mL	Li et al. [87]
Albuminuria	Solution	Smartphone	~61 nM.	Zhao et al. [95]
VOCs	Paper-based	Wearable sensor, naked eye	7.04 mg/m^3^ (1.8 ppm)	Mengyao et al. [101]
VOCs	Paper-based	Smartphone	-	Hyungi et al. [102]
*Giardia lamblia* cyst	Cellulose membrane	Smartphone	12 cysts/10 mL	Koydemir et al. [106]
Tetracycline (TC)	Paper strips	Smartphone	50 nM	Zhang et al. [116]
Water	Solution	Smartphone	1.2%	Liang et al. [121]

## Data Availability

Not applicable.

## References

[1] Nishi K., Isobe S.-I., Zhu Y., Kiyama R. (2015). Fluorescence-based bioassays for the detection and evaluation of food materials. Sensors.

[2] Camarca A., Varriale A., Capo A., Pennacchio A., Calabrese A., Giannattasio C., Murillo Almuzara C., D’Auria S., Staiano M. (2021). Emergent Biosensing Technologies Based on Fluorescence Spectroscopy and Surface Plasmon Resonance. Sensors.

[3] Nath P., Hamadna S.S., Karamchand L., Foster J., Kopelman R., Amar J.G., Ray A. (2021). Intracellular detection of singlet oxygen using fluorescent nanosensors. Analyst.

[4] Ray A., Ranieri P., Karamchand L., Yee B., Foster J., Raoul K. (2017). Real-Time Monitoring of Intracellular Chemical Changes in Response to Plasma Irradiation. Plasma Med..

[5] Ray A., Kopelman R. (2013). Hydrogel nanosensors for biophotonic imaging of chemical analytes. Nanomedicine.

[6] Strianese M., Staiano M., Ruggiero G., Labella T., Pellecchia C., D’Auria S. (2012). Fluorescence-based biosensors. Methods Mol. Biol..

[7] Pham H., Hoseini Soflaee M., Karginov A.V., Miller L.W. (2022). Förster resonance energy transfer biosensors for fluorescence and time-gated luminescence analysis of rac1 activity. Sci. Rep..

[8] Komatsu N., Terai K., Imanishi A., Kamioka Y., Sumiyama K., Jin T., Okada Y., Nagai T., Matsuda M. (2018). A platform of BRET-FRET hybrid biosensors for optogenetics, chemical screening, and in vivo imaging. Sci. Rep..

[9] Liu L., He F., Yu Y., Wang Y. (2020). Application of FRET Biosensors in Mechanobiology and Mechanopharmacological Screening. Front. Bioeng. Biotechnol..

[10] Yu L., Lei Y., Ma Y., Liu M., Zheng J., Dan D., Gao P. (2021). A Comprehensive Review of Fluorescence Correlation Spectroscopy. Front. Phys..

[11] Hendrickson O.D., Taranova N.A., Zherdev A.V., Dzantiev B.B., Eremin S.A. (2020). Fluorescence Polarization-Based Bioassays: New Horizons. Sensors.

[12] Liu L., Zhou X., Wilkinson J.S., Hua P., Song B., Shi H. (2017). Integrated optical waveguide-based fluorescent immunosensor for fast and sensitive detection of microcystin-LR in lakes: Optimization and Analysis. Sci. Rep..

[13] Squire K., Kong X., LeDuff P., Rorrer G.L., Wang A.X. (2018). Photonic crystal enhanced fluorescence immunoassay on diatom biosilica. J. Biophotonics.

[14] Sharma A., Khan R., Catanante G., Sherazi T.A., Bhand S., Hayat A., Marty J.L. (2018). Designed Strategies for Fluorescence-Based Biosensors for the Detection of Mycotoxins. Toxins.

[15] Nath P., Kopelman R., Foster J., Amar J., Ray A. (2021). Fluorescent nanosensors for detection of intracellular singlet oxygen during plasma therapy. Proc. SPIE.

[16] Ray A., Lee Y.-E.K., Elbez R., Kopelman R. (2012). Targeted nanosensor aided three-dimensional pH mapping in tumor spheroids using two-photon microscopy. Proc. SPIE.

[17] Ray A., Koo Lee Y.-E., Epstein T., Kim G., Kopelman R. (2011). Two-photon nano-PEBBLE sensors: Subcellular pH measurements. Analyst.

[18] Ray A., Kopelman R., Rajian J.R., Wang X., Lee Y.-E.K. (2012). Lifetime-based photoacoustic oxygen sensing in vivo. J. Biomed. Opt..

[19] Mandracchia B., Hua X., Guo C., Son J., Urner T., Jia S. (2020). Fast and accurate sCMOS noise correction for fluorescence microscopy. Nat. Commun..

[20] Ceylan Koydemir H., Ray A. (2020). Mobile Diagnostic Devices for Digital Transformation in Personalized Healthcare. Diagnostics.

[21] Nath P., Kabir M.A., Doust S.K., Ray A. (2021). Diagnosis of Herpes Simplex Virus: Laboratory and Point-of-Care Techniques. Infect. Dis. Rep..

[22] Shin Y.-H., Teresa Gutierrez-Wing M., Choi J.-W. (2021). Review—Recent Progress in Portable Fluorescence Sensors. J. Electrochem. Soc..

[23] Pham A.T.T., Tohl D., Wallace A., Hu Q., Li J., Reynolds K.J., Tang Y. (2022). Developing a fluorescent sensing based portable medical open-platform—A case study for albuminuria measurement in chronic kidney disease screening and monitoring. Sens. Bio-Sens. Res..

[24] Sueker M., Stromsodt K., Gorji H.T., Vasefi F., Khan N., Schmit T., Varma R., Mackinnon N., Sokolov S., Akhbardeh A. (2021). Handheld Multispectral Fluorescence Imaging System to Detect and Disinfect Surface Contamination. Sensors.

[25] Vietz C., Schütte M.L., Wei Q., Richter L., Lalkens B., Ozcan A., Tinnefeld P., Acuna G.P. (2019). Benchmarking Smartphone Fluorescence-Based Microscopy with DNA Origami Nanobeads: Reducing the Gap toward Single-Molecule Sensitivity. ACS Omega.

[26] Lee W.-I., Park Y., Park J., Shrivastava S., Son Y.-M., Choi H.-J., Lee J., Jeon B., Lee H., Lee N.-E. (2019). A smartphone fluorescence imaging-based mobile biosensing system integrated with a passive fluidic control cartridge for minimal user intervention and high accuracy. Lab Chip.

[27] Arias-Alpízar K., Sánchez-Cano A., Prat-Trunas J., de la Serna Serna E., Alonso O., Sulleiro E., Sánchez-Montalvá A., Diéguez A., Baldrich E. (2022). Malaria quantitative POC testing using magnetic particles, a paper microfluidic device and a hand-held fluorescence reader. Biosens. Bioelectron..

[28] Ruiz-Vega G., Arias-Alpízar K., de la Serna E., Borgheti-Cardoso L.N., Sulleiro E., Molina I., Fernàndez-Busquets X., Sánchez-Montalvá A., Del Campo F.J., Baldrich E. (2020). Electrochemical POC device for fast malaria quantitative diagnosis in whole blood by using magnetic beads, Poly-HRP and microfluidic paper electrodes. Biosens. Bioelectron..

[29] Loeffelholz M.J., Tang Y.-W. (2021). Detection of SARS-CoV-2 at the point of care. Bioanalysis.

[30] Song Q., Sun X., Dai Z., Gao Y., Gong X., Zhou B., Wu J., Wen W. (2021). Point-of-care testing detection methods for COVID-19. Lab Chip.

[31] Greenwood D.A., Grady M. (2020). Healthcare Professional Perceptions of Blood Glucose Meter Features That Support Achievement of Self-Management Goals Recommended by Clinical Practice Guidelines. J. Diabetes Sci. Technol..

[32] Nath P., Arun R.K., Chanda N. (2015). Smart gold nanosensor for easy sensing of lead and copper ions in solution and using paper strips. RSC Adv..

[33] Nath P., Arun R.K., Chanda N. (2014). A paper based microfluidic device for the detection of arsenic using a gold nanosensor. RSC Adv..

[34] McMullin T.S., Bamber A.M., Bon D., Vigil D.I., Van Dyke M. (2018). Exposures and Health Risks from Volatile Organic Compounds in Communities Located near Oil and Gas Exploration and Production Activities in Colorado (U.S.A.). Int. J. Environ. Res. Public Health.

[35] Leonard J., Koydemir H.C., Koutnik V.S., Tseng D., Ozcan A., Mohanty S.K. (2022). Smartphone-enabled rapid quantification of microplastics. J. Hazard. Mater. Lett..

[36] Mudanyali O., Oztoprak C., Tseng D., Erlinger A., Ozcan A. (2010). Detection of waterborne parasites using field-portable and cost-effective lensfree microscopy. Lab Chip.

[37] Kriss R., Pieper K.J., Parks J., Edwards M.A. (2021). Challenges of Detecting Lead in Drinking Water Using at-Home Test Kits. Environ. Sci. Technol..

[38] Reddy R.R., Rodriguez G.D., Webster T.M., Abedin M.J., Karim M.R., Raskin L., Hayes K.F. (2020). Evaluation of arsenic field test kits for drinking water: Recommendations for improvement and implications for arsenic affected regions such as Bangladesh. Water Res..

[39] Kost G.J., Tran N.K., Louie R.F. (2008). Point-of-Care Testing: Principles, Practice, and Critical-Emergency-Disaster Medicine. Encyclopedia of Analytical Chemistry.

[40] Kruk M.E., Gage A.D., Arsenault C., Jordan K., Leslie H.H., Roder-DeWan S., Adeyi O., Barker P., Daelmans B., Doubova S.V. (2018). High-quality health systems in the Sustainable Development Goals era: Time for a revolution. Lancet Glob. Health.

[41] Vashist S.K. (2017). Point-of-Care Diagnostics: Recent Advances and Trends. Biosensors.

[42] St John A., Price C.P. (2013). Economic Evidence and Point-of-Care Testing. Clin. Biochem. Rev..

[43] Ventola C.L. (2015). The antibiotic resistance crisis: Part 1: Causes and threats. P T.

[44] Prestinaci F., Pezzotti P., Pantosti A. (2015). Antimicrobial resistance: A global multifaceted phenomenon. Pathog. Glob. Health.

[45] Kadri S.S. (2020). Key Takeaways From the U.S. CDC’s 2019 Antibiotic Resistance Threats Report for Frontline Providers. Crit. Care Med..

[46] Nath P., Kabir A., Khoubafarin Doust S., Kreais Z.J., Ray A. (2020). Detection of Bacterial and Viral Pathogens Using Photonic Point-of-Care Devices. Diagnostics.

[47] Peri A.M., Stewart A., Hume A., Irwin A., Harris P.N.A. (2021). New Microbiological Techniques for the Diagnosis of Bacterial Infections and Sepsis in ICU Including Point of Care. Curr. Infect. Dis. Rep..

[48] Tsalik E.L., Bonomo R.A., Fowler V.G. (2018). New Molecular Diagnostic Approaches to Bacterial Infections and Antibacterial Resistance. Annu. Rev. Med..

[49] Kim J., Park J.-Y., Park Y.-J., Park S.-Y., Lee M.-S., Koo C. (2020). A portable and high-sensitivity optical sensing system for detecting fluorescently labeled enterohaemorrhagic Escherichia coli Shiga toxin 2B-subunit. PLoS ONE.

[50] Zhou Q., Pan J., Mo L., Luo Z., Qin Z., Dai Z., Yi C. (2022). Fluorescent on-site detection of multiple pathogens using smartphone-based portable device with paper-based isothermal amplification chip. Mikrochim. Acta.

[51] Papadakis G., Pantazis A.K., Fikas N., Chatziioannidou S., Tsiakalou V., Michaelidou K., Pogka V., Megariti M., Vardaki M., Giarentis K. (2022). Portable real-time colorimetric LAMP-device for rapid quantitative detection of nucleic acids in crude samples. Sci. Rep..

[52] Huang J., Zhong Y., Li W., Wang W., Li C., Wang A., Yan H., Wan Y., Li J. (2021). Fluorescent and Opt-Electric Recording Bacterial Identification Device for Ultrasensitive and Specific Detection of Microbials. ACS Sens..

[53] Shin J., Yoon T., Park J., Park K.S. (2022). Sensitive and simultaneous detection of hygiene indicator bacteria using an enhanced CRISPR/Cas system in combination with a portable fluorescence detector. Sens. Actuators B Chem..

[54] Sheini A. (2021). A point-of-care testing sensor based on fluorescent nanoclusters for rapid detection of septicemia in children. Sens. Actuators B Chem..

[55] Xie H., Mire J., Kong Y., Chang M., Hassounah H.A., Thornton C.N., Sacchettini J.C., Cirillo J.D., Rao J. (2012). Rapid point-of-care detection of the tuberculosis pathogen using a BlaC-specific fluorogenic probe. Nat. Chem..

[56] Moustakas M. (2021). The Role of Metal Ions in Biology, Biochemistry and Medicine. Materials.

[57] Lakatos B., Szentmihályi K., Vinkler P., Balla J., Balla G. (2004). The role of essential metal ions in the human organism and their oral supplementation to the human body in deficiency states. Orv. Hetil..

[58] Martínez-Mier E.A. (2011). Fluoride: Its Metabolism, Toxicity, and Role in Dental Health. J. Evid. Based. Complement. Altern. Med..

[59] Duffin S., Duffin M., Grootveld M. (2022). Revisiting Fluoride in the Twenty-First Century: Safety and Efficacy Considerations. Front. Oral Health.

[60] Zheng X., Cheng W., Ji C., Zhang J., Yin M. (2020). Detection of metal ions in biological systems: A review. Rev. Anal. Chem..

[61] Dean K.M., Qin Y., Palmer A.E. (2012). Visualizing metal ions in cells: An overview of analytical techniques, approaches, and probes. Biochim. Biophys. Acta.

[62] Zhang L., Gao X., Chen X., Zhao M., Wu H., Liu Y. (2022). A smartphone integrated ratiometric fluorescent sensor for point-of-care testing of fluoride ions. Anal. Bioanal. Chem..

[63] Yang Q., Jia C., Chen Q., Du W., Wang Y., Zhang Q. (2017). A NIR fluorescent probe for the detection of fluoride ions and its application in in vivo bioimaging. J. Mater. Chem. B.

[64] Dey S., Kumar A., Mondal P.K., Chopra D., Roy R., Jindani S., Ganguly B., Mayya C., Bhatia D., Jain V.K. (2022). Ultrasensitive colorimetric detection of fluoride and arsenate in water and mammalian cells using recyclable metal oxacalixarene probe: A lateral flow assay. Sci. Rep..

[65] Zhang S., Sun M., Yan Y., Yu H., Yu T., Jiang H., Zhang K., Wang S. (2017). A turn-on fluorescence probe for the selective and sensitive detection of fluoride ions. Anal. Bioanal. Chem..

[66] Liu X., Liu X., Shen Y., Gu B. (2020). A Simple Water-Soluble ESIPT Fluorescent Probe for Fluoride Ion with Large Stokes Shift in Living Cells. ACS Omega.

[67] Yan L., Li D., Le Y., Dong P., Liu L. (2022). Phenothiazine-based fluorescent probe for fluoride ions and its applications in rapid detection of endemic disease. Dye. Pigment..

[68] Ma Y., Mou Q., Yan P., Yang Z., Xiong Y., Yan D., Zhang C., Zhu X., Lu Y. (2021). A highly sensitive and selective fluoride sensor based on a riboswitch-regulated transcription coupled with CRISPR-Cas13a tandem reaction. Chem. Sci..

[69] Nakata E., Nazumi Y., Yukimachi Y., Uto Y., Hori H., Morii T. (2014). Self-Assembled Fluorescent Nanoprobe for the Detection of Fluoride Ions in Aqueous Solutions. Bull. Chem. Soc. Jpn..

[70] Wu X., Wang H., Yang S., Tian H., Liu Y., Sun B. (2019). Highly Sensitive Ratiometric Fluorescent Paper Sensors for the Detection of Fluoride Ions. ACS Omega.

[71] Ke B., Chen W., Ni N., Cheng Y., Dai C., Dinh H., Wang B. (2013). A fluorescent probe for rapid aqueous fluoride detection and cell imaging. Chem. Commun. (Camb).

[72] Chansaenpak K., Kamkaew A., Weeranantanapan O., Suttisintong K., Tumcharern G. (2018). Coumarin Probe for Selective Detection of Fluoride Ions in Aqueous Solution and Its Bioimaging in Live Cells. Sensors.

[73] Yu X., Yang L., Zhao T., Zhang R., Yang L., Jiang C., Zhao J., Liu B., Zhang Z. (2017). Multicolorful ratiometric-fluorescent test paper for determination of fluoride ions in environmental water. RSC Adv..

[74] Zhang C., Kim J.P., Creer M., Yang J., Liu Z. (2017). A smartphone-based chloridometer for point-of-care diagnostics of cystic fibrosis. Biosens. Bioelectron..

[75] Zhang J., Li Y., Chai F., Li Q., Wang D., Liu L., Tang B.Z., Jiang X. (2022). Ultrasensitive point-of-care biochemical sensor based on metal-AIEgen frameworks. Sci. Adv..

[76] Rinschen M.M., Ivanisevic J., Giera M., Siuzdak G. (2019). Identification of bioactive metabolites using activity metabolomics. Nat. Rev. Mol. Cell Biol..

[77] Shine B., Guha N. (2020). The use of biochemical analysis for diagnosis and management. Oxford Textb. Med..

[78] Treacy O., Brown N.N., Dimeski G. (2019). Biochemical evaluation of kidney disease. Transl. Androl. Urol..

[79] Zadhoush F., Sadeghi M., Pourfarzam M. (2015). Biochemical changes in blood of type 2 diabetes with and without metabolic syndrome and their association with metabolic syndrome components. J. Res. Med. Sci..

[80] Guo L., Chen S., Yu Y.-L., Wang J.-H. (2021). A Smartphone Optical Device for Point-of-Care Testing of Glucose and Cholesterol Using Ag NPs/UiO-66-NH2-Based Ratiometric Fluorescent Probe. Anal. Chem..

[81] Wang B., Zhou X.-Q., Li L., Li Y.-X., Yu L.-P., Chen Y. (2022). Ratiometric fluorescence sensor for point-of-care testing of bilirubin based on tetraphenylethylene functionalized polymer nanoaggregate and rhodamine B. Sens. Actuators B Chem..

[82] Johnson S.E., Sherding R.G. (2006). Diseases of the Liver and Biliary Tract. Saunders Man. Small Anim. Pract..

[83] VanWagner L.B., Green R.M. (2015). Evaluating elevated bilirubin levels in asymptomatic adults. JAMA.

[84] Meher S., Mishra T.S., Sasmal P.K., Rath S., Sharma R., Rout B., Sahu M.K. (2015). Role of Biomarkers in Diagnosis and Prognostic Evaluation of Acute Pancreatitis. J. Biomark..

[85] Kylänpää-Bäck M.L., Kemppainen E., Puolakkainen P. (2002). Trypsin-based laboratory methods and carboxypeptidase activation peptide in acute pancreatitis. JOP.

[86] Heinrich H.C., Gabbe E.E., Icagić F. (1979). Immunoreactive serum trypsin in diseases of the pancreas. Klin. Wochenschr..

[87] Li H., Yang M., Kong D., Jin R., Zhao X., Liu F., Yan X., Lin Y., Lu G. (2019). Sensitive fluorescence sensor for point-of-care detection of trypsin using glutathione-stabilized gold nanoclusters. Sens. Actuators B Chem..

[88] Melman Y., Wells P.K., Katz E., Smutok O. (2022). A universal nanostructured bioanalytical platform for NAD+-dependent enzymes based on the fluorescent output reading with a smartphone. Talanta.

[89] Wells P.K., Smutok O., Guo Z., Alexandrov K., Katz E. (2023). Fluorometric biosensing of α-amylase using an artificial allosteric biosensor immobilized on nanostructured interface. Talanta.

[90] Wells P.K., Smutok O., Guo Z., Alexandrov K., Katz E. (2022). Nanostructured Interface Loaded with Chimeric Enzymes for Fluorimetric Quantification of Cyclosporine A and FK506. Anal. Chem..

[91] Ninomiya T., Perkovic V., de Galan B.E., Zoungas S., Pillai A., Jardine M., Patel A., Cass A., Neal B., Poulter N. (2009). Albuminuria and kidney function independently predict cardiovascular and renal outcomes in diabetes. J. Am. Soc. Nephrol..

[92] Butt L., Unnersjö-Jess D., Höhne M., Edwards A., Binz-Lotter J., Reilly D., Hahnfeldt R., Ziegler V., Fremter K., Rinschen M.M. (2020). A molecular mechanism explaining albuminuria in kidney disease. Nat. Metab..

[93] Shibata K. (2014). Urine 3-hydroxykynurenine is higher during the postovulatory phase than in the preovulatory phase indicating a higher vitamin B6 requirement. Biosci. Biotechnol. Biochem..

[94] Birková A., Valko-Rokytovská M., Hubková B., Zábavníková M., Mareková M. (2021). Strong Dependence between Tryptophan-Related Fluorescence of Urine and Malignant Melanoma. Int. J. Mol. Sci..

[95] Zhao X., Zheng W., Qin T., Du X., Lei Y., Lv T., Zhou M., Xu Z., Wang L., Liu B. (2022). An anti-interference fluorescent probe for point-of-care diagnosis of albuminuria. Sens. Actuators B Chem..

[96] Gray W.B., Shimshack J.P. (2011). The Effectiveness of Environmental Monitoring and Enforcement: A Review of the Empirical Evidence. Rev. Environ. Econ. Policy.

[97] Gałuszka A., Migaszewski Z.M., Namieśnik J. (2015). Moving your laboratories to the field—Advantages and limitations of the use of field portable instruments in environmental sample analysis. Environ. Res..

[98] Donaire-Gonzalez D., Valentín A., de Nazelle A., Ambros A., Carrasco-Turigas G., Seto E., Jerrett M., Nieuwenhuijsen M.J. (2016). Benefits of Mobile Phone Technology for Personal Environmental Monitoring. JMIR Mhealth Uhealth.

[99] Tiele A., Esfahani S., Covington J. (2018). Design and Development of a Low-Cost, Portable Monitoring Device for Indoor Environment Quality. J. Sens..

[100] Galstyan V., D’Arco A., Di Fabrizio M., Poli N., Lupi S., Comini E. (2021). Detection of volatile organic compounds: From chemical gas sensors to terahertz spectroscopy. Rev. Anal. Chem..

[101] Zhang M., Gao L., Zhao X., Duan Y., Liao Y., Han T. (2022). Fabricating a hydroxynaphthalene benzophenone Schiff base into a wearable fluorescent sensor for point-of-care sensing of volatile organic compounds. Dye. Pigment..

[102] Kim H., Choi S.-K., Ahn J., Yu H., Min K., Hong C., Shin I.-S., Lee S., Lee H., Im H. (2021). Kaleidoscopic fluorescent arrays for machine-learning-based point-of-care chemical sensing. Sens. Actuators B Chem..

[103] Stillo F., MacDonald Gibson J. (2017). Exposure to Contaminated Drinking Water and Health Disparities in North Carolina. Am. J. Public Health.

[104] Allaire M., Wu H., Lall U. (2018). National trends in drinking water quality violations. Proc. Natl. Acad. Sci. USA.

[105] Mueller J.T., Gasteyer S. (2021). The widespread and unjust drinking water and clean water crisis in the United States. Nat. Commun..

[106] Koydemir H.C., Gorocs Z., Tseng D., Cortazar B., Feng S., Chan R.Y.L., Burbano J., McLeod E., Ozcan A. (2015). Rapid imaging, detection and quantification of Giardia lamblia cysts using mobile-phone based fluorescent microscopy and machine learning. Lab Chip.

[107] Tok S., de Haan K., Tseng D., Usanmaz C.F., Ceylan Koydemir H., Ozcan A. (2019). Early detection of E. coli and total coliform using an automated, colorimetric and fluorometric fiber optics-based device. Lab Chip.

[108] Göröcs Z., Tamamitsu M., Bianco V., Wolf P., Roy S., Shindo K., Yanny K., Wu Y., Koydemir H.C., Rivenson Y. (2018). A deep learning-enabled portable imaging flow cytometer for cost-effective, high-throughput, and label-free analysis of natural water samples. Light Sci. Appl..

[109] Ceylan Koydemir H., Feng S., Liang K., Nadkarni R., Benien P., Ozcan A. (2017). Comparison of supervised machine learning algorithms for waterborne pathogen detection using mobile phone fluorescence microscopy. Nanophotonics.

[110] Koydemir H.C., Gorocs Z., McLeod E., Tseng D., Ozcan A. (2015). Field portable mobile phone based fluorescence microscopy for detection of Giardia lamblia cysts in water samples. Proc. SPIE.

[111] Müller V., Sousa J.M., Ceylan Koydemir H., Veli M., Tseng D., Cerqueira L., Ozcan A., Azevedo N.F., Westerlund F. (2018). Identification of pathogenic bacteria in complex samples using a smartphone based fluorescence microscope. RSC Adv..

[112] Lin L., Yang H., Xu X. (2022). Effects of Water Pollution on Human Health and Disease Heterogeneity: A Review. Front. Environ. Sci..

[113] Igboama W.N., Hammed O.S., Fatoba J.O., Aroyehun M.T., Ehiabhili J.C. (2022). Review article on impact of groundwater contamination due to dumpsites using geophysical and physiochemical methods. Appl. Water Sci..

[114] Ahmad F., Zhu D., Sun J. (2021). Environmental fate of tetracycline antibiotics: Degradation pathway mechanisms, challenges, and perspectives. Environ. Sci. Eur..

[115] Dai Y., Liu M., Li J., Yang S., Sun Y., Sun Q., Wang W., Lu L., Zhang K., Xu J. (2020). A review on pollution situation and treatment methods of tetracycline in groundwater. Sep. Sci. Technol..

[116] Zhang J., Shi G. (2022). Rational design of MoS(2) QDs and Eu(3+) as a ratiometric fluorescent probe for point-of-care visual quantitative detection of tetracycline via smartphone-based portable platform. Anal. Chim. Acta.

[117] Guo Y., Zhao W. (2019). Nanomaterials for luminescent detection of water and humidity. Analyst.

[118] Deng Q., Li Y., Wu J., Liu Y., Fang G., Wang S., Zhang Y. (2012). Highly sensitive fluorescent sensing for water based on poly(m-aminobenzoic acid). Chem. Commun..

[119] Ye C., Qin Y., Huang P., Chen A., Wu F.-Y. (2018). Facile synthesis of carbon nanodots with surface state-modulated fluorescence for highly sensitive and real-time detection of water in organic solvents. Anal. Chim. Acta.

[120] Wei J., Li H., Yuan Y., Sun C., Hao D., Zheng G., Wang R. (2018). A sensitive fluorescent sensor for the detection of trace water in organic solvents based on carbon quantum dots with yellow fluorescence. RSC Adv..

[121] Liang H., Li Y., Lin B., Yu Y., Wang Y., Zhang L., Cao Y., Guo M. (2022). Multicolor fluorescent probe for visual point-of-care detection of water via a smartphone. Microchem. J..

[122] Alam M.W., Wahid K.A., Goel R.K., Lukong K.E. (2019). Development of a low-cost and portable smart fluorometer for detecting breast cancer cells. Biomed. Opt. Express.

[123] Shin Y.H.O., Gutierrez-Wing M.T., Choi J.-W. (2021). A Portable Fluorometer with Multiple Excitation LEDs. ECS Meet. Abstr..

[124] Ozcan A. (2014). Mobile phones democratize and cultivate next-generation imaging, diagnostics and measurement tools. Lab Chip.

[125] Skolrood L., Wang Y., Zhang S., Wei Q. (2022). Single-molecule and particle detection on true portable microscopy platforms. Sens. Actuators Rep..

[126] Trofymchuk K., Glembockyte V., Grabenhorst L., Steiner F., Vietz C., Close C., Pfeiffer M., Richter L., Schütte M.L., Selbach F. (2021). Addressable nanoantennas with cleared hotspots for single-molecule detection on a portable smartphone microscope. Nat. Commun..

[127] You L. (2020). Superconducting nanowire single-photon detectors for quantum information. Nanophotonics.

[128] Yokogawa S., Oshiyama I., Ikeda H., Ebiko Y., Hirano T., Saito S., Oinoue T., Hagimoto Y., Iwamoto H. (2017). IR sensitivity enhancement of CMOS Image Sensor with diffractive light trapping pixels. Sci. Rep..

[129] Ray A., Esparza S., Wu D., Hanudel M.R., Joung H.-A., Gales B., Tseng D., Salusky I.B., Ozcan A. (2020). Measurement of serum phosphate levels using a mobile sensor. Analyst.

[130] Wang H., Heintzmann R., Diederich B. (2021). The power in your pocket—Uncover smartphones for use as cutting-edge microscopic instruments in science and research. Adv. Opt. Technol..

[131] Kabir M.A., Kharel A., Malla S., Kreis Z.J., Nath P., Wolfe J.N., Hassan M., Kaur D., Sari-Sarraf H., Tiwari A.K. (2022). Automated detection of apoptotic versus nonapoptotic cell death using label-free computational microscopy. J. Biophotonics.

[132] Ray A., Lee Y.-E.K., Kim G., Kopelman R. (2012). Two-photon fluorescence imaging super-enhanced by multishell nanophotonic particles, with application to subcellular pH. Small.

[133] Wei Q., Acuna G., Kim S., Vietz C., Tseng D., Chae J., Shir D., Luo W., Tinnefeld P., Ozcan A. (2017). Plasmonics Enhanced Smartphone Fluorescence Microscopy. Sci. Rep..

[134] Mei Z., Tang L. (2017). Surface-Plasmon-Coupled Fluorescence Enhancement Based on Ordered Gold Nanorod Array Biochip for Ultrasensitive DNA Analysis. Anal. Chem..

[135] Semeniak D., Cruz D.F., Chilkoti A., Mikkelsen M.H. (2022). Plasmonic Fluorescence Enhancement in Diagnostics for Clinical Tests at Point-of-Care: A Review of Recent Technologies. Adv. Mater..

[136] Bauch M., Toma K., Toma M., Zhang Q., Dostalek J. (2014). Plasmon-Enhanced Fluorescence Biosensors: A Review. Plasmonics.

[137] Jeong Y., Kook Y.-M., Lee K., Koh W.-G. (2018). Metal enhanced fluorescence (MEF) for biosensors: General approaches and a review of recent developments. Biosens. Bioelectron..

[138] Hoang T.X., Phan L.M., Vo T.A., Cho S. (2021). Advanced Signal-Amplification Strategies for Paper-Based Analytical Devices: A Comprehensive Review. Biomedicines.

[139] Ding X., Ge D., Yang K.-L. (2014). Colorimetric protease assay by using gold nanoparticles and oligopeptides. Sens. Actuators B Chem..

[140] Xiao W., Xiong Y., Li Y., Chen Z., Li H. (2023). Non-Enzymatically Colorimetric Bilirubin Sensing Based on the Catalytic Structure Disruption of Gold Nanocages. Sensors.

[141] Khlebtsov B.N., Tumskiy R.S., Burov A.M., Pylaev T.E., Khlebtsov N.G. (2019). Quantifying the Numbers of Gold Nanoparticles in the Test Zone of Lateral Flow Immunoassay Strips. ACS Appl. Nano Mater..

[142] Miller B.S., Thomas M.R., Banner M., Kim J., Chen Y., Wei Q., Tseng D.K., Göröcs Z.S., Ozcan A., Stevens M.M. (2022). Sub-picomolar lateral flow antigen detection with two-wavelength imaging of composite nanoparticles. Biosens. Bioelectron..

[143] Anker J.N., Kopelman R. (2003). Magnetically modulated optical nanoprobes. Appl. Phys. Lett..

[144] Zhang Y., Ouyang M., Ray A., Liu T., Kong J., Bai B., Kim D., Guziak A., Luo Y., Feizi A. (2019). Computational cytometer based on magnetically modulated coherent imaging and deep learning. Light Sci. Appl..

[145] Xiao Z., Darwish G.H., Susumu K., Medintz I.L., Algar W.R. (2022). Prototype Smartphone-Based Device for Flow Cytometry with Immunolabeling via Supra-nanoparticle Assemblies of Quantum Dots. ACS Meas. Sci. Au.

[146] Xue Y., Liu C., Andrews G., Wang J., Ge Y. (2022). Recent advances in carbon quantum dots for virus detection, as well as inhibition and treatment of viral infection. Nano Converg..

[147] Zoghi S., Rahmandoust M. (2022). A novel technique to overcome fluid flow influence in carbon quantum dots/paper-based analytical devices. Sci. Rep..

[148] Manivannan S., Ponnuchamy K. (2020). Quantum dots as a promising agent to combat COVID-19. Appl. Organomet. Chem..

[149] Rabiee N., Ahmadi S., Soufi G.J., Hekmatnia A., Khatami M., Fatahi Y., Iravani S., Varma R.S. (2022). Quantum dots against SARS-CoV-2: Diagnostic and therapeutic potentials. J. Chem. Technol. Biotechnol..

[150] Mousavi S.M., Hashemi S.A., Yari Kalashgrani M., Omidifar N., Lai C.W., Vijayakameswara Rao N., Gholami A., Chiang W.-H. (2022). The Pivotal Role of Quantum Dots-Based Biomarkers Integrated with Ultra-Sensitive Probes for Multiplex Detection of Human Viral Infections. Pharmaceuticals.

[151] Azam N., Najabat Ali M., Javaid Khan T. (2021). Carbon Quantum Dots for Biomedical Applications: Review and Analysis. Front. Mater..

[152] Sun K., Yang Y., Zhou H., Yin S., Qin W., Yu J., Chiu D.T., Yuan Z., Zhang X., Wu C. (2018). Ultrabright Polymer-Dot Transducer Enabled Wireless Glucose Monitoring via a Smartphone. ACS Nano.

[153] Gupta R., Peveler W.J., Lix K., Algar W.R. (2019). Comparison of Semiconducting Polymer Dots and Semiconductor Quantum Dots for Smartphone-Based Fluorescence Assays. Anal. Chem..

[154] Ferrero F.J., Valledor M., Campo J.C., López A., Llano-Suárez P., Fernández-Arguelles M.T., Costa-Fernández J.M., Soldado A. (2020). Portable Instrument for Monitoring Environmental Toxins Using Immobilized Quantum Dots as the Sensing Material. Appl. Sci..

[155] Zhao Q., Zhang C., Liu S., Liu Y., Zhang K.Y., Zhou X., Jiang J., Xu W., Yang T., Huang W. (2015). Dual-emissive Polymer Dots for Rapid Detection of Fluoride in Pure Water and Biological Systems with Improved Reliability and Accuracy. Sci. Rep..

[156] Manmana Y., Kubo T., Otsuka K. (2021). Recent developments of point-of-care (POC) testing platform for biomolecules. TrAC Trends Anal. Chem..

[157] Foudeh A.M., Fatanat Didar T., Veres T., Tabrizian M. (2012). Microfluidic designs and techniques using lab-on-a-chip devices for pathogen detection for point-of-care diagnostics. Lab Chip.

[158] Arshavsky-Graham S., Segal E., Bahnemann J., Grünberger A. (2022). Lab-on-a-Chip Devices for Point-of-Care Medical Diagnostics BT—Microfluidics in Biotechnology.

[159] Wu J., Dong M., Rigatto C., Liu Y., Lin F. (2018). Lab-on-chip technology for chronic disease diagnosis. Npj Digit. Med..

[160] Sachdeva S., Davis R.W., Saha A.K. (2021). Microfluidic Point-of-Care Testing: Commercial Landscape and Future Directions. Front. Bioeng. Biotechnol..

[161] Al-Aqbi Z.T., Albukhaty S., Zarzoor A.M., Sulaiman G.M., Khalil K.A.A., Belali T., Soliman M.T.A. (2021). A Novel Microfluidic Device for Blood Plasma Filtration. Micromachines.

[162] Asawakarn S., Pimpin A., Jeamsaksiri W., Sripumkhai W., Jitsamai W., Taweethavonsawat P., Piyaviriyakul P. (2023). Application of a novel rectangular filtering microfluidic device for microfilarial detection. Front. Vet. Sci..

[163] Lo S.-J., Kuan C.-M., Hung M.-W., Fu Y., Yeh J.A., Yao D.-J., Cheng C.-M. (2018). A Simple Imaging Device for Fluorescence-Relevant Applications. Micromachines.

[164] Chalam K.V., Chamchikh J., Gasparian S. (2022). Optics and Utility of Low-Cost Smartphone-Based Portable Digital Fundus Camera System for Screening of Retinal Diseases. Diagnostics.

[165] Ji Y., Kim J., Kim J., Lee M., Noh J., Jeong T., Shin J., Kim J., Heo Y., Cho U. Reliability characterization of advanced CMOS image sensor (CIS) with 3D stack and in-pixel DTI. Proceedings of the 2018 IEEE International Reliability Physics Symposium (IRPS).

[166] Filipovic L., Lahlalia A. (2018). (Invited) System-on-Chip Sensor Integration in Advanced CMOS Technology. ECS Trans..

[167] Smuck M., Odonkor C.A., Wilt J.K., Schmidt N., Swiernik M.A. (2021). The emerging clinical role of wearables: Factors for successful implementation in healthcare. Npj Digit. Med..

[168] Lee S.M., Lee D. (2020). Healthcare wearable devices: An analysis of key factors for continuous use intention. Serv. Bus..

[169] Vijayan V., Connolly J.P., Condell J., McKelvey N., Gardiner P. (2021). Review of Wearable Devices and Data Collection Considerations for Connected Health. Sensors.

[170] Sood R., Kaur P., Sharma S., Mehmuda A., Kumar A. IoT Enabled Smart Wearable Device—Sukoon. Proceedings of the 2018 Fourteenth International Conference on Information Processing (ICINPRO).

[171] Gv D.R., Eshwar S., Dhanamjayulu C., Ragul M., Senkathir M. IoT Enabled Predicting Wearables for the Pandemic. Proceedings of the 2021 Innovations in Power and Advanced Computing Technologies (i-PACT).

[172] Rao S. (2021). IoT Enabled Wearable Device for COVID Safety and Emergencies. Int. J. Interact. Mob. Technol..

[173] Wan J., Al-Awlaqi M.A.A.H., Li M., O’Grady M., Gu X., Wang J., Cao N. (2018). Wearable IoT enabled real-time health monitoring system. EURASIP J. Wirel. Commun. Netw..

[174] Alonso O., Franch N., Canals J., Arias-Alpízar K., de la Serna E., Baldrich E., Diéguez A. (2020). An internet of things-based intensity and time-resolved fluorescence reader for point-of-care testing. Biosens. Bioelectron..

